# Network Mendelian randomization analysis deciphers protein pathways linking type 2 diabetes and gastrointestinal disease

**DOI:** 10.1111/dom.16087

**Published:** 2024-11-26

**Authors:** Jiawei Geng, Xixian Ruan, Xing Wu, Xuejie Chen, Tian Fu, Dipender Gill, Stephen Burgess, Jie Chen, Jonas F. Ludvigsson, Susanna C. Larsson, Xue Li, Zhongyan Du, Shuai Yuan

**Affiliations:** 1 Zhejiang Key Laboratory of Blood-Stasis-Toxin Syndrome, Zhejiang Chinese Medical University, Hangzhou, 310053, China; 2 Department of Big Data in Health Science School of Public Health, Center of Clinical Big Data and Analytics of The Second Affiliated Hospital, Zhejiang University School of Medicine, Hangzhou, China; 3 Department of Gastroenterology, The Third Xiangya Hospital, Central South University, Changsha, China; 4 Department of Epidemiology and Biostatistics, School of Public Health, Imperial College London, London SW7 2BX, UK; 5 MRC Biostatistics Unit, University of Cambridge, Cambridge, UK; 6 Department of Public Health and Primary Care, University of Cambridge, Cambridge, UK; 7 Department of Medical Epidemiology and Biostatistics, Karolinska Institutet, Stockholm, Sweden; 8 Department of Pediatrics, Orebro University Hospital, Orebro, Sweden; 9 Department of Medicine, Celiac Disease Center at Columbia University Medical Center, New York, New York, USA; 10 Unit of Cardiovascular and Nutritional Epidemiology, Institute of Environmental Medicine, Karolinska Institutet, Stockholm, Sweden; 11 Unit of Medical Epidemiology, Department of Surgical Sciences, Uppsala University, 10 Uppsala, Sweden; 12 Zhejiang Engineering Research Center for "Preventive Treatment" Smart Health of Traditional Chinese Medicine, Zhejiang Chinese Medical University, Hangzhou, 310053, China; 13 School of Basic Medical Sciences, Zhejiang Chinese Medical University, Hangzhou, 310053, China; 14 Department of surgery, Perelman School of Medicine at the University of Pennsylvania, Philadelphia, PA, USA

**Keywords:** drug target, gastrointestinal diseases, Mendelian randomization, protein biomarker, type 2 diabetes

## Abstract

**Aims:**

The molecular mechanisms underlying the association between type 2 diabetes (T2D) and gastrointestinal (GI) disease are unclear. To identify protein pathways, we conducted a two-stage network Mendelian randomization (MR) study.

**Materials and methods:**

Genetic instruments for T2D were obtained from a large-scale summary-level genome-wide meta-analysis. Genetic associations with blood protein levels were obtained from three genome-wide association studies on plasma proteins (i.e., the deCODE study as the discovery, and the UKB-PPP and Fenland studies as the replication). Summary-level data on 10 GI diseases were derived from genome-wide meta-analysis of the UK Biobank and FinnGen. MR and colocalization analyses were performed. Pathways were constructed according to the directionality of total and indirect effects and corresponding proportional mediation was estimated. Druggability assessments were conducted across four databases to prioritize protein mediators.

**Results:**

Genetic liability to T2D was associated with 69 proteins in the discovery protein dataset after multiple testing corrections. All associations were replicated at the nominal significance level. Among T2D-associated proteins, genetically predicted levels of 9 proteins were associated with at least one of the GI diseases. Genetically predicted levels of SULT2A1 (odds ratio=1.98, 95% CI 1.80-2.18), and ADH1B (odds ratio=2.05, 95% CI 1.43-2.94) were associated with cholelithiasis and cirrhosis, respectively. SULT2A1 and cholelithiasis (PH4=0.996), and ADH1B and cirrhosis (PH4=0.931) have strong colocalization support, accounting for the mediation proportion of 72.8% (95% CI 45.7-99.9) and 42.9% (95% CI 15.5-70.4), respectively.

**Conclusions:**

The study identified some proteins mediating T2D-GI disease associations, which provided biological insights into the underlying pathways.

## 1 Introduction

Type 2 diabetes mellitus (T2D) affects around 480 million population globally ^1^. As the fastest growing metabolic disorders, T2D imposes a significant disease burden worldwide ^2^. Gastrointestinal (GI) disease is more prevalent in patients with T2D compared to the general population, and the presence of GI disease substantially deteriorates life quality ^3^. Although accumulating evidence supports T2D as a risk factor for developing a wide range of GI diseases ^4–7^, the underlying biological pathways remain unclear.

Circulating proteins play important roles in biological process and have high potentials as drug targets ^8,9^. Unravelling protein links between T2D and GI disease may provide crucial insights into disease pathogenesis understanding, early disease prevention, and target drug development. Blood protein alteration has been observed in patients with T2D ^10–12^ and implicated in GI diseases as well ^13–16^.

Mendelian randomization (MR) using randomly allocated genetic variants as instrument variables has the advantages of minimizing confounding and reversal causation ^17^. MR approach has been widely used to understand disease pathophysiology ^18^. Recent MR studies have identified several plasma proteins associated with T2D ^19,20^ and some GI diseases ^21–23^; however, the shared protein basis between T2D and GI diseases has been scarcely been studied. We here applied MR analysis under the two-stage network framework to identify the intermediate proteins linking T2D and GI diseases.

## 2 Materials and methods

### 2.1 Study design

**Figure 1:**
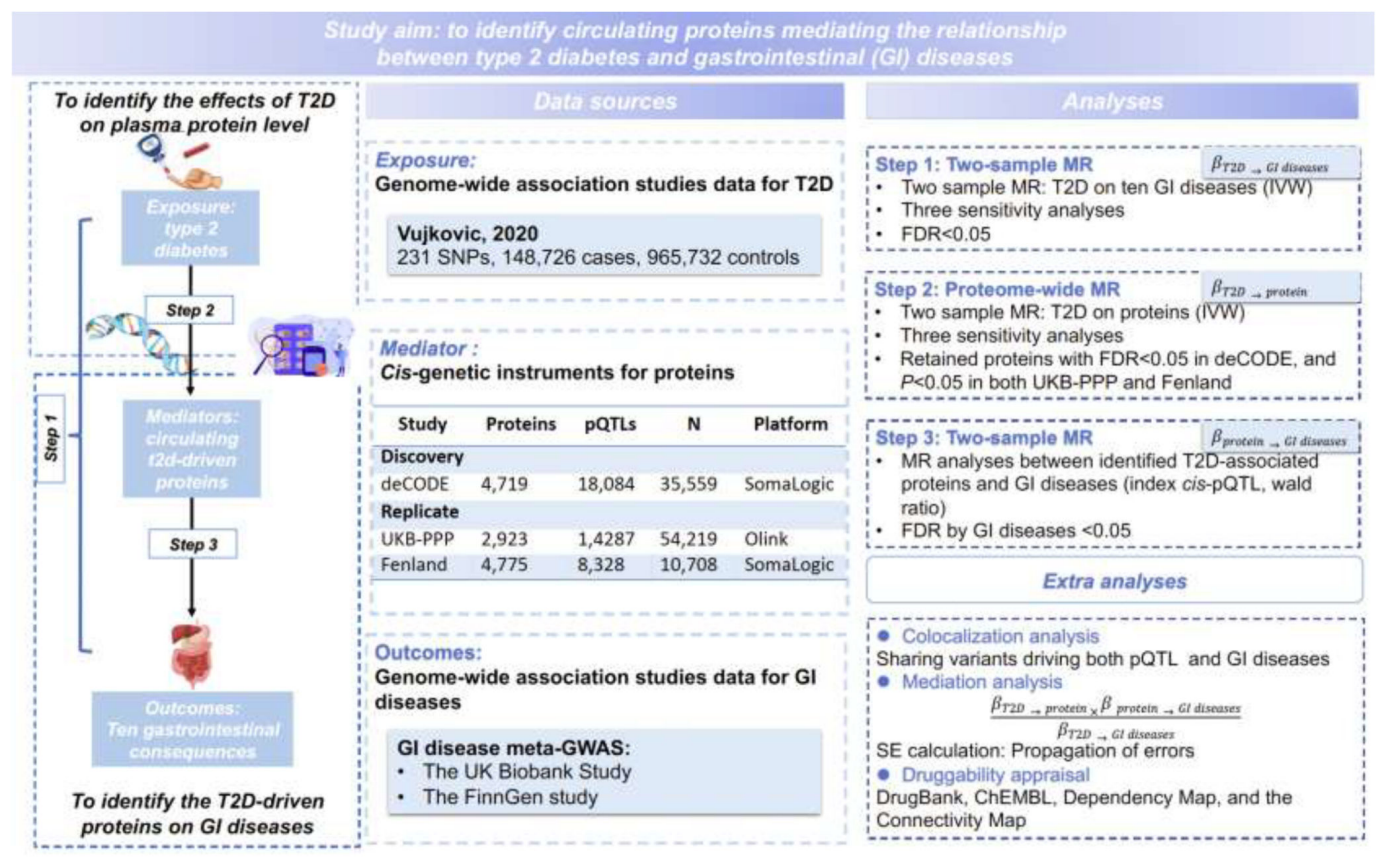
Study flow chart. GI, gastrointestinal; SNP, single nucleotide polymorphisms; T2D, type 2 diabetes; IVW, inverse variance weighted.

The study design is presented in Figure 1. Based on our previous explorations ^4^, we included 10 GI diseases associated with T2D in this study. There are three steps of two-stage MR analysis. First, we examined T2D-GI disease associations. In the second step, we conducted a proteome-wide MR to explore the proteins associated with genetic liability to T2D. To increase the reliability, only proteins survived after the multiple testing correction and consistently replicated in replication datasets were regarded as putative T2D-associated proteins and retained for subsequent analyses. In the last step, we estimated the associations between T2D-associated proteins and GI disease risk. Several extra analyses including genetic colocalization analysis, mediation calculation, and druggability assessment were conducted to prioritize protein mediators.

### 2.2 Study population and data sources

#### Genetic instruments for T2D

Genetic instruments for T2D were obtained from the Vujkovic *et al* GWAS incorporating 148,726 cases and 965,732 non-cases of European ancestry ^24^. We selected genetic variants (e.g., single-nucleotide polymorphisms [SNPs]) associated with T2D at the genome-wide significance level (*P* <5×10^-8^) and a low linkage disequilibrium (defined as *r*^2^<0.001) as the instrumental variables. To minimize horizontal pleiotropy, we excluded variants in the *FTO* gene ^4,25^ via searching in PhenoScanner V2 ^26^.

#### Blood protein data sources

We obtained GWAS data on blood protein levels from three large-scale studies without sample overlap. The discovery analysis was based on the deCODE protein genetic database that assessed genetic variants’ associations with 4,719 unique plasma proteins measured in 35,559 Icelanders ^27^. We further used two replication data sources from the UK Biobank Pharma Proteomics Project (UKB-PPP) where 2,923 unique proteins were profiled in 54,219 participants ^28^ as well as from the Fenland study where genetic associations were calculated for 4,775 proteins in 10,708 Caucasian ^29^. Both the deCODE and Fenland studies profiled blood protein data using the SomaScan version 4 assay (SomaLogic) while the UKB-PPP used Olink Explore 3072 PEA. Regarding genetic instrumental variable selection for proteins, we obtained the index *cis*-acting variant defined as the SNP located within 1 Mb upstream or downstream of the transcription start site of the protein encoding gene and with the smallest *p* value ^30^.

##### Data sources for GI diseases

The study included 10 GI diseases including four upper GI diseases (gastroesophageal reflux disease, gastric ulcer, acute gastritis, chronic gastritis), two lower GI diseases (irritable bowel syndrome, diverticular disease), and four hepato-biliary and pancreatic diseases (cholelithiasis, cholecystitis, nonalcoholic fatty liver disease, and cirrhosis). Summary-level data on these outcomes were obtained from the UK Biobank and FinnGen R9 release. The UK Biobank is an ongoing cohort study, which recruited half a million of participant across the United Kingdom between 2006 and 2010 aged over 40 ^31^. Participants with GI diseases were ascertained by International Classification of Diseases (ICD)-8, ICD-9 and ICD-10. Summary-level data on the 10 GI diseases were based on individuals of European ancestry. GWAS analysis was performed by the Lee Lab (Seoul National University, Seoul, Republic of Korea; https://www.leelabsg.org/resources) with the adjustment of sex, birth year, genotyping batch, and first four principal components. The FinnGen study included Finnish adults. GI diseases were ascertained by the ICD codes from nationwide health registers. GWAS analysis was adjusted for sex, age, genotyping batch, and the first ten genetic principal components ^32^. Detailed information on two studies is presented in **Additional file 1**: Table S1. We performed the GWAS meta-analysis of the UK Biobank and FinnGen R9 using the inverse-variance fixed-effects method by METAL software with genomic control correction ^33^. To increase the statistical power, our MR analyses were based on these genome-wide meta-analysis data.

### 2.3 Statistical analyses

#### MR analysis

Data harmonization was performed based on both effect and other alleles. Due low levels of missingness, we did not replace SNPs that were not available in one of the datasets with proxies. The inverse variance weighted (IVW) and the Wald ratio methods were used as the primary analysis depending on the number of SNPs included in the analysis. To test the robustness of the results, MR-Egger ^34^, weighted median ^35^, and weighted mode ^36^ analyses were performed as the sensitivity analyses. Cochran’s Q statistic was calculated to measure the heterogeneity. The IVW analysis was guided by the heterogeneity test. Specifically, the fixed-effects IVW method was applied when the *P*-value for Cochran’s Q statistic was ≥0.05; otherwise, the random-effects IVW method was used.^37^ Horizontal pleiotropy was assessed by MR-Egger intercept test ^34^. The false discovery rate (FDR) based on *Benjamini-Hochberg* approach was used for multiple testing correction. A significance level of FDR-correctred *P* <0.05 was considered significant. All tests were two-sided, and TwoSampleMR package in R software were used to perform analyses.

#### Colocalization analysis

To investigate whether the protein and GI disease share a causal variant ^38^, we conducted genetic Bayesian colocalization analysis using the coloc R package. For each protein, we included SNPs within the 1Mb region around the lead *cis*-pQTL in the primary analysis, and included SNPs within 500kb in the supplementary analysis. Five exclusive hypotheses are as follows: 1) no association with either protein or GI outcomes (P_H0_); 2) one causal variant for protein only (P_H1_); 3) one causal variant for GI disease only (P_H2_); 4) two distinct causal variants for protein and GI disease were associated (P_H3_); 5) a shared causal variant for both protein and GI disease (P_H4_). The prior probability for the causal variant associated with trait 1 only (P_1_), trait 2 only (P_2_), both trait 1 and 2 (P_12_) were set as 1×10^-4^, 1×10^-4^, and 1×10^-5^. We defined the P_H4_ ≥0.8 as strong evidence of colocalization, 0.5≤P_H4_<0.8 as medium evidence, and P_H4_ <0.5 as low evidence.

#### Mediation analysis

We performed the mediation analysis according to directional consistency of between total effect (*β*_T2D-GI disease_) and indirect effect (*β*_T2D-protein_ × *β*_Protein-GI disease_) among the associations reaching the FDR significance level in both step 2 (*β*_T2D-protein_) and step 3 (*β*_Protein-GI disease_) MR analyses. We then estimated the proportion mediated of protein in the T2D-GI disease association. In detail, the product of coefficient method was applied to estimate the indirect effect ^39^. The proportion was calculated by multiplying the estimate of T2D-protein association and the estimate of protein-GI disease association (indirect effect=*β*_T2D-protein_ × *β*_protein-GI disease_) then dividing by the estimate of T2D-GI disease association (*β*_T2D-GI disease_). The propagation method was used to calculate the CI ^39^.

#### Appraisal of druggability

We assessed the druggability of potential protein mediators with the aim of prioritizing therapeutic targets. This assessment was conducted with data from the DrugBank ^40^, ChEMBL ^41^, Dependency Map, and the Connectivity Map (https://clue.io/repurposing-app). Treating protein as the drug target, we documented the information on the drug name, drug indication, and development process in the pipeline were documented. We characterized the therapeutic development process into four categories: 1) approved; 2) in clinical trials; 3) preclinical; and 4) druggable ^21^.

##### Data availability

All data used in the study were from the publicly available summary-level datasets. All studies obtained the ethical permission from the relevant ethical committees, and participants provided informed consent. The genome-wide meta-analysis data on GI disease are deposited in https://osf.io/kxehz/?view_only=e24c3bb0a59b4d89aaa226ea86566262.

## 3 Results

### 3.1 Genetic liability to T2D in relation to GI disease

**Figure 2:**
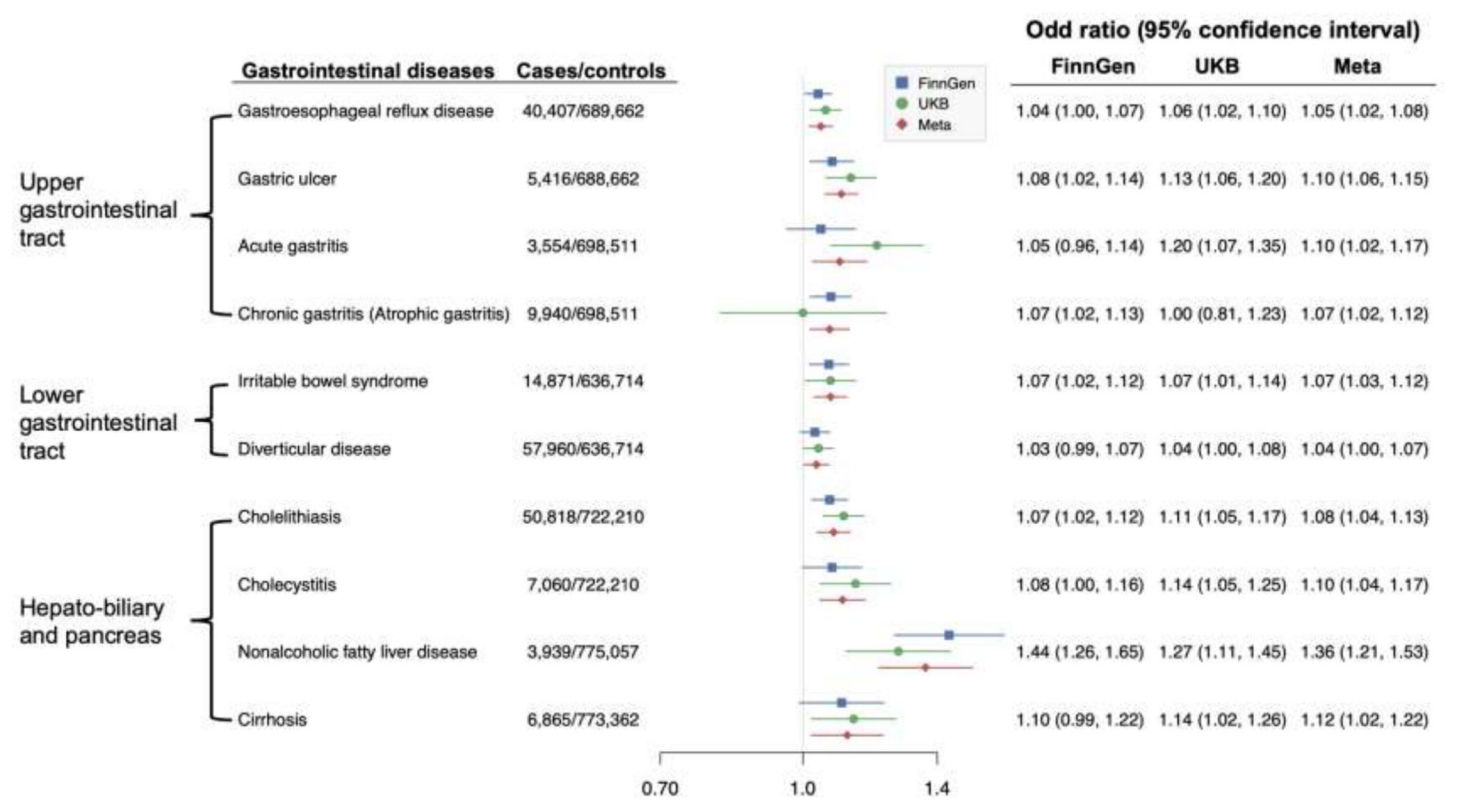
Associations of genetically predicted type 2 diabetes with gastrointestinal diseases. The ORs were scaled to 1-unit increase in log-transformed OR of type 2 diabetes. CI, confidence interval; OR, odd ratio.

Genetic liability to T2D was associated with all ten GI diseases after FDR correction, with ORs ranging from 1.05 (95% CI 1.02, 1.08, *P*=0.002) for gastroesophageal reflux disease to 1.36 (95% CI 1.21, 1.53, *P*=2.94×10^-7^) for nonalcoholic fatty liver disease (Figure 2). The associations remained overall consistent in the sensitivity analyses **(Additional file 1**: Table S2).

### 3.2 Genetic liability to T2D in relation to protein levels

**Figure 3:**
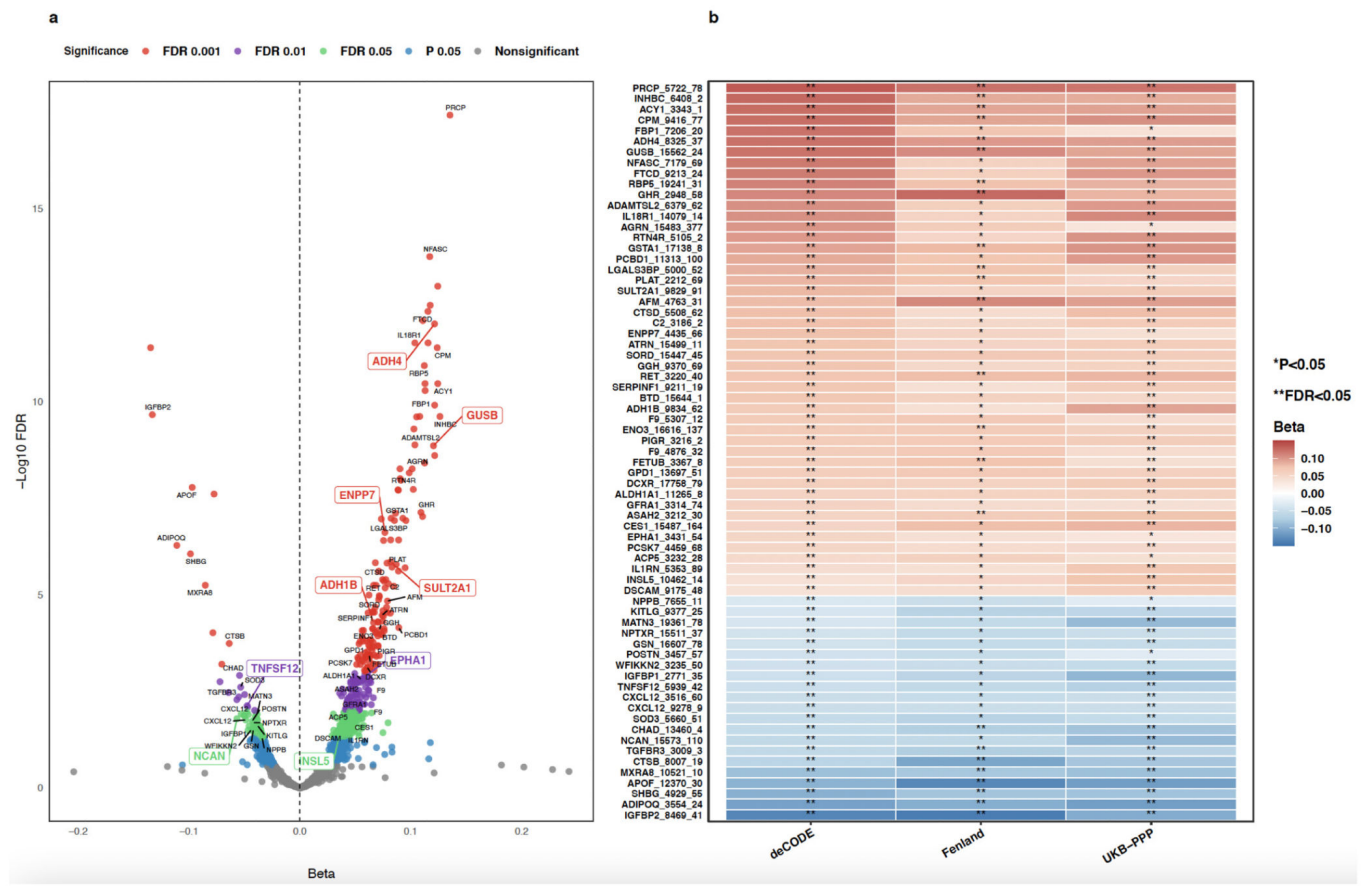
**(a) Volcano plots of MR association between genetically predicted T2D and circulating protein levels in the discovery dataset (deCODE).** The labeled proteins are 69 out of 464 discovered proteins with FDR<0.05 that directionally consistent and maintained nominal significant (*P*<0.05) in the UKB-PPP and Fenland datasets. **(b) Heatmap of associations replicated using protein data from UKB-PPP, and Fenland.** The association with a *P* value <0.05, but FDR corrected *P* value ≥0.05 was labeled with *, while FDR corrected *P* value <0.05 was labeled as ** in the heatmap. FDR performed among all proteins in each dataset. ADH1B, Alcohol dehydrogenase 1B; ADH4, Alcohol dehydrogenase 4; ENPP7, Ectonucleotide pyrophosphatase/phosphodiesterase family member 7; EPHA1, Ephrin type-A receptor 1; FDR, false discovery rate; GI, gastrointestinal; GUSB, Beta-glucuronidase; INSL5, Insulin-like peptide INSL5; NCAN, Neurocan core protein; SULT2A1, Bile salt sulfotransferase; T2D, type 2 diabetes; TNFSF12, Tumor necrosis factor ligand superfamily member 12.

The association between genetic liability to T2D and levels of blood proteins were examined in three protein genome-wide association datasets. In the discovery dataset, a total of 464 of 4,907 proteins was associated with genetic liability to T2D after FDR correction (Figure 3A, and **Additional file 1**: Table S3). Among these, 69 associations were directionally replicated in UKB-PPP and Fenland (*P*<0.05, Figure 3B, Additional file 1: Table S4, and S5).

### 3.3 T2D-associated proteins in relation to GI diseases

We then performed two-sample MR analysis to estimate the associations between 69 T2D-driven proteins and GI diseases. Given some proteins had no suitable instruments, 60 proteins with *cis*-acting (protein quantitative trait loci) pQTL (*P* <5×10^-8^, *r*^2^<0.01) were included. Eleven pairs of putative T2D driven protein associated with GI diseases were identified using the deCODE database. This included three proteins (ADH4 [alcohol dehydrogenase 1B], ENPP7 [ectonucleotide pyrophosphatase/phosphodiesterase family member 7], and SULT2A1 [bile salt sulfotransferase]) associated with cholelithiasis, two proteins (ADH1B [alcohol dehydrogenase 1B] and NCAN [neurocan core protein]) associated with cirrhosis, two proteins (GUSB [beta-glucuronidase] and NCAN) associated with NAFLD, two proteins (EPHA1 [ephrin type-A receptor 1] and SULT2A1 [bile salt sulfotransferase]) with cholecystitis, one protein (TNFSF12 [tumor necrosis factor ligand superfamily member 12]) associated with diverticular disease, and one protein (INSL5 [insulin-like peptide 5]) associated with gastric ulcer (Table 1 and **Additional file 1**: Table S6). All these pairs were directionally replicated in the UKB-PPP database, while ADH1B-cirrhosis and EPHA1-cholecystitis did not reach the significant level (**Additional file 1**: Table S7). In the Fenland study, all identified pairs reached nominal significant level (*P*<0.05) except for ENPPT (the analysis could not be performed due to lack of available variants) **(Additional file 1**: Table S8).

### 3.4 Colocalization analysis

SULT2A1 (P_H4_=0.996) and ADH1B (P_H4_=0.931) had high support colocalization with cholelithiasis and cirrhosis in the deCODE dataset, respectively. SULT2A1 maintained strong colocalization using other two data sources (P_H4_=0.998 in UKB-PPP, and P_H4_=0.961 in Fenland). While strong colocalization for ADH1B was replicated in Fenland study (P_H4_=0.933), evidence was weaker in UKB-PPP (P_H4_=0.181). Moderate colocalization were observed between GUSB and NAFLD, and INSL5 and gastric ulcer (0.8>P_H4_>0.7) (Table 1, and **Additional file 1**: Table S9).

### 3.5 Mediation analysis

**Figure 4:**
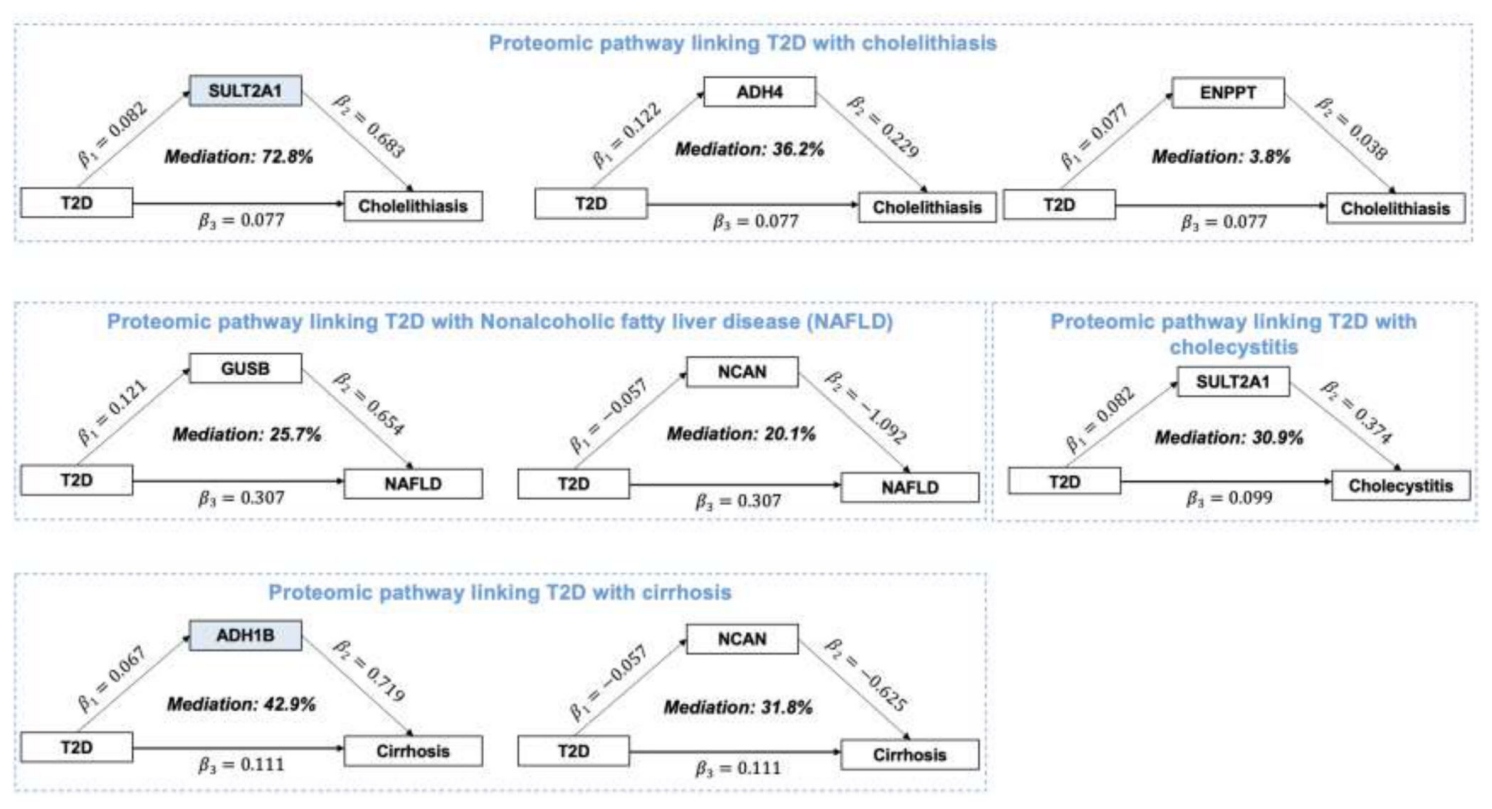
Proteomic mediators of the associations between T2D and GI diseases. The association was based on the deCODE protein dataset. Blue coloured protein encoding genes (SULT2A1 and ADH1B) demonstrating high evidence of colocalization with associated GI diseases (PH4≥0.8). ADH1B, Alcohol dehydrogenase 1B; GI, gastrointestinal; SULT2A1, Bile salt sulfotransferase; T2D, type 2 diabetes.

Among eleven protein-GI disease pairs, mediation effect was not found between EPHA1 and cholecystitis, INSL5 and gastric ulcer, TNFSF12 and diverticular disease due to directionality inconsistency. SULT2A1 mediated around 72.8% (95% CI, 45.7%, 99.9%) of the association between T2D and cholelithiasis. This mediation was observed in UKB-PPP and Fenland albeit attenuated. ADH1B mediated 42.9% (95% CI, 15.5%, 70.4%) of the association between T2D and cirrhosis using deCODE protein data and 45.9% (95% CI 0%, 92.6%) using the Fenland protein study. (Figure 4, and **Additional file 1**: Table S10). There were some other mediations with moderate effects including ADH4-cholelithiasis (36.2%, 95% CI, 13.6%, 58.8%), ENPP7-cholithiasis (3.8%, 95% CI, 1.3%, 6.3%), GUSB-NAFLD (25.7%, 95% CI, 10.9%, 40.4%), NCAN-cirrhosis (31.8%, 95% CI, 10.5%, 53.1%), NCAN-NAFLD (20.1%, 95% CI, 7.4%, 32.9%), and SULT2A1-cholecystitis (30.9%, 95% CI, 10.0%, 51.8%) **(Additional file 1**: Table S10).

### 3.6 Druggability analysis

We assessed druggability for 9 protein mediators in T2D-GI disease association. As summarized in **Additional file 1**: Table S11, SULT2A1 and ADH1B were listed as druggable target with wide implications in cancer and chronic pain treatment. However, neither of them has been recognized to treat GI diseases.

## 4 Discussion

### 4.1 Principal findings

The study employed a two-stage network MR analysis and studied the potential roles of blood proteins mediating association between T2D and a wide range of GI diseases. Putative causal plasma proteins associated withT2D were screened among more than 4,000 proteins. We identified 69 proteins associated with genetically predicted T2D. We then established the MR associations between these proteins and GI disease risk. Along with genetic colocalization and mediation construction, we highlighted two potential protein pathways linking T2D and GI diseases. SULT2A1 mediated around 72.8% of T2D-cholelithiasis association and ADH1B mediated 42.9% of T2D-cirrhosis association. These findings provided biological insights into the underlying pathways for T2D and GI disease associations.

### 4.2 Comparison with previous studies

The results from the present study are in line with previous findings. Some identified T2D-associated proteins were reported including well-studied proteins like SHBG (sex hormone-binding globulin) ^42^, C2 (complement C2) ^43^, and MXRA8 (matrix remodeling associated 8) ^44^. A prior MR-based association study identified T2D was associated with SULT2A1 (bile salt sulfotransferase) ^44^. Supported by earlier observations, reduced level of plasma TNFSF12 (TWEAK) ^45^ and elevated level of GUSB (β-glucuronidase) ^46^ were reported in prevalent T2D. ENPP7 has been identified as novel biomarkers associated with glycaemic deterioration in T2D ^47^. Two alcohol dehydrogenase (ADH) enzymes, ADH1B and ADH4 were identified as the putative T2D associated proteins. ADH protein regulates alcohol metabolism and may influence the development of T2D. The association between ADH4 and incident T2D was identified in a previous cohort study ^46^, but the effect of T2D on the ADH1B and ADH4 is less explored. However, considering the complex biological pathways due to possible pleiotropy, further study is warranted to disclose the underlying chain of T2D-to-protein effect.

A recent study revealed shared genetic mechanisms between T2D and gastroesophageal reflux disease, irritable bowel syndrome, peptic ulcer disease (i.e, gastric ulcer), and gastritis-duodenitis (i.e, acute gastritis)^48^. The study found multiple genes and biological pathways are shared between T2D and various GI disorders, involving autoimmune, viral, and proinflammatory-mediated mechanisms ^48^. Our study reveals underlying connections between T2D and GI diseases from a protein perspective by integrating human genetic and proteomics data. These findings offer valuable insights for clinical translation and potential opportunities for drug target repurposing and development.

Our findings offer protein insights into the association between the protein links to T2D and GI diseases. Among 11 identified protein-GI disease pairs, the evidence of colocalization for most associations were weak, indicating the possible confounding from linkage disequilibrium. The strongest signal was SULT2A1 that may play a role in the development of cholelithiasis among T2D. SULT2A1, known as bile salt sulfotransferase, belongs to hydroxysteroid sulfotransferase (SULT) family. SULT is highly expressed in liver, and metabolically active or hormonally responsive tissues outside the liver ^49^. The association between T2D and SULT2A1 has been reported in an MR study using the proteomics source from 5,438 elderly Icelanders.^44^ Several large GWAS also identified susceptibility loci at SULT2A1 associated with risk of cholelithiasis (gallstone disease) ^29,50,51^. Cholelithiasis is featured as the formation of one or more gallstones in the gallbladder and bile acids dysregulation is one of the established mechanisms ^52^. Insulin resistance can decrease the expression of bile acid synesthetic enzymes and increase the gallstone susceptibility ^53^. Thus, alternation of SULT2A1 among T2D patients may change the hepatic sulfation of bile acid, bile acid metabolism, and in turn increase the risk of developing cholelithiasis ^51^.

The putative role of ADH1B in linking T2D to cirrhosis was also noted. ADH1B is involved in ethanol metabolic pathway ^54^ and has potential role in insulin resistance ^55,56^. Molecular studies suggested that elevated expression of ADH1B decreased the expression of an intracellular lipid transporter FABP4, which can stimulate β-cells to secrete insulin to maintain glucose homeostasis ^55^. ADH1B is predominately expressed in liver, and accumulating evidence suggested its association with cirrhosis, especially alcohol-related liver cirrhosis ^57,58^. However, no previous studies have revealed that this protein is associated with excessive cirrhosis caused by T2D. Nevertheless, the rs1229984 variant in the *ADH1B* has been strongly linked to T2D, potentially altering alcohol consumption and resulting in T2D development ^59^. We could not rule out that the observed mediating effect of ADH1B in the association between T2D and cirrhosis might be driven by an upstream effect of alcohol consumption on T2D. Thus, future studies are needed to warrant our findings.

### 4.3 Strengths

This study is the first study to explore the protein pathway between T2D and GI disease and provided insights into the complex pathophysiological process of GI disease comorbidities in T2D. The MR study design has the advantages of reducing the confounding and reverse causation and facilitate the causal inference. Another notable strength of study is that we used multiple data sources and conducted a series sensitivity and replication analyses to validate our findings. In addition, combined evidence from colocalization analysis, we were able to rule out the possible bias caused by linkage disequilibrium. The stringent replication criteria enhanced the reliability of our findings.

### 4.4 Limitations

The study also has some limitations. First, some proteins might be inadvertently neglected due to lack of *cis*-pQTL signals. Second, the current study used data only from European ancestry. Although it can minimize the population stratification bias, it decreases the generalizability of our findings to other populations. Third, pleiotropic effect could not be fully ruled out. To minimize the bias, we removed the SNPs near the *FTO* gene that has been association with obesity, a shared risk factor between T2D and GI diseases.^4^ Fourth, although we used genome-wide meta-analysis of two large cohorts for the GI diseases, statistical power might still be inadequate for weak-to-moderate associations for some outcomes with a small number of cases. Further study using emerging larger GWAS is warranted.

## 5 Conclusions

In summary, the study identified many circulating proteins associated with T2D. SULT2A1 and ADH1B were suggested as vital protein biomarkers mediating the association between T2D and cholelithiasis and the association between T2D and cirrhosis, respectively.

## List of abbreviations

ADH1B: Alcohol dehydrogenase 1B
ADH4: Alcohol dehydrogenase 4
C2: Complement C2
CI: Confidence interval
ENPP7: Ectonucleotide pyrophosphatase/phosphodiesterase family member 7
EPHA1: Ephrin type-A receptor 1
FDR: False discovery rate
GI: Gastrointestinal
GUSB: Beta-glucuronidase
GWAS: Genome-wide association studies
INSL5: Insulin-like peptide 5
ICD: International Classification of Diseases
IVW: Inverse variance weighted
UKB-PPP: UK Biobank Pharma Proteomics Project
MR: Mendelian randomization
MXRA8: Matrix remodeling associated 8
NCAN: Neurocan core protein
NAFLD: Nonalcoholic fatty liver disease
OR: Odd ratio
pQTL: Protein quantitative trait loci
SHBG: Sex hormone-binding globulin
SULT2A1: Bile salt sulfotransferase
SNP: Single-nucleotide polymorphisms
T2D: Type 2 diabetes
TNFSF12: Tumor necrosis factor ligand superfamily member 12

## Supplementary Material

Supplementary material

Additional file

## Figures and Tables

**Table 1:** Associations of putative type 2 diabetes-associated proteins with gastrointestinal diseases.

Gene ^a^	GI disease	Discovery (deCODE)			Replication (UKB-PPP)			Replication (Fenland)		
		OR (95% CI)	*P*	P_H4_^b^	OR (95% CI)	*P*	P_H4_^b^	OR (95% CI)	*P*	P_H4_^b^
*ADH1B*	Cirrhosis	2.05 (1.43,2.94)	8.96E-05	**0.931**	1.81 (0.82,4.03)	0.145	0.181	3.49 (1.87,6.51)	8.96E-05	**0.933**
*ADH4*	Cholelithiasis	1.26 (1.10,1.43)	0.001	0.363	NA	NA	NA	1.20 (1.05,1.38)	8.54E-03	0.107
*ENPP7*	Cholelithiasis	1.04 (1.02,1.06)	4.19E-04	0.028	NA	NA	NA	NA	NA	NA
*EPHA1*	Cholecystitis	0.87 (0.80,0.95)	0.001	0.013	1.05 (0.87,1.25)	0.629	0.009	0.99 (0.94,1.03)	0.542	0.016
*GUSB*	NAFLD	1.92 (1.39,2.67)	8.61E-05	0.017	2.10 (1.45,3.04)	8.61E-05	0.723	1.79 (1.31,2.44)	2.51E-04	0.562
*INSL5*	Gastric ulcer	0.39 (0.23,0.65)	3.18E-04	0.774	0.74 (0.63,0.87)	3.18E-04	0.800	0.60 (0.46,0.79)	3.18E-04	**0.817**
*NCAN*	Cirrhosis	0.54 (0.44,0.65)	1.85E-10	<0.001	0.48 (0.38,0.61)	1.09E-09	<0.001	0.48 (0.39,0.61)	1.85E-10	<0.001
*NCAN*	NAFLD	0.34 (0.26,0.43)	1.34E-18	0.009	0.30 (0.22,0.41)	1.07E-14	0.001	0.28 (0.21,0.37)	1.34E-18	0.008
*SULT2A1*	Cholecystitis	1.45 (1.17,1.81)	0.001	0.589	1.23 (1.09,1.39)	8.03E-04	0.638	1.35 (1.13,1.62)	1.03E-03	0.593
*SULT2A1*	Cholelithiasis	1.98 (1.80,2.18)	1.28E-45	**0.996**	1.47 (1.39,1.55)	1.28E-45	**0.998**	1.64 (1.52,1.77)	1.28E-36	**0.961**
*TNFSF12*	Diverticular disease	1.11 (1.05,1.17)	2.78E-04	0.001	1.15 (1.08,1.22)	1.08E-05	0.001	1.05 (1.01,1.08)	8.55E-03	<0.001

a Presented results reached FDR<0.05 in MR analysis based on deCODE database.

b P_H4_ values were based on colocalization analysis under ±1000kb window.

NA indicates the absence of *cis*-pQTL. ADH1B, Alcohol dehydrogenase 1B; ADH4, Alcohol dehydrogenase 4; CI, confidence interval; ENPP7, Ectonucleotide pyrophosphatase/phosphodiesterase family member 7; EPHA1, Ephrin type-A receptor 1; FDR, false discovery rate; GI, gastrointestinal; GUSB, Beta-glucuronidase; INSL5, Insulin-like peptide INSL5; NCAN, Neurocan core protein; NAFLD; Nonalcoholic fatty liver disease; OR, odd ratio; SULT2A1, Bile salt sulfotransferase; TNFSF12, Tumor necrosis factor ligand superfamily member 12.

## Data Availability

All data used in the current study were obtained from publicly released GWAS summary statistics. The single-nucleotide polymorphisms (SNP) of type 2 diabetes (T2D) can be extracted from the study Vujkovic M et al. (2020, PMID32541925). The pQTL GWAS summary for deCODE, Fenland, and UKB-PPP study used in the analyses can be downloaded from https://www.decode.com/summarydata/; www.omicscience.org/apps/pgwas, and http://ukb-ppp.gwas.eu, respectively. Summary-level data on these outcomes were obtained from the UK Biobank (https://www.leelabsg.org/resources), FinnGen R9 release (https://www.finngen.fi/fi), and the genome-wide meta-analysis data are deposited in https://osf.io/kxehz/?view_only=e24c3bb0a59b4d89aaa226ea86566262. Database for drug and drug target are available at DrugBank (https://go.drugbank.com/), Dependency Map (https://depmap.org/portal/), Connectivity Map (https://repo-hub.broadinstitute.org/repurposing-app), and ChEMBL (https://www.ebi.ac.uk/chembl/).
