# Supplementary material for: Network Mendelian randomization analysis deciphers protein pathways linking type 2 diabetes and gastrointestinal disease
Source: Diabetes Obes Metab. Author manuscript; Available in PMC 2025 Feb 1. (PMC7617254; doi:10.1111/dom.16087)
Supplement: Additional file [file EMS201614-supplement-Additional_file.docx]

**Additional file 1:**

**Supplementary Tables**

**Table S1.** Dataset description.

**Table S2.** Association of type 2 diabetes with ten gastrointestinal diseases.

**Table S3.** Association of type 2 diabetes with deCODE plasma protein.

**Table S4.** Association of type 2 diabetes with UKB-PPP plasma protein.

**Table S5.** Association of type 2 diabetes with Fenland plasma protein.

**Table S6.** Association of putative type 2 diabetes-associated proteins in deCODE with gastrointestinal diseases.

**Table S7.** Association of putative type 2 diabetes-associated proteins in UKB-PPP with gastrointestinal diseases.

**Table S8.** Association of putative type 2 diabetes-associated proteins in Fenland with gastrointestinal diseases.

**Table S9.** Colocalization analysis of identified protein-gastrointestinal pairs in deCODE, UKB-PPP, and Fenland.

**Table S10.** Mediation analysis of identified protein between type 2 diabetes and gastrointestinal diseases in deCODE, UKB-PPP, and Fenland.

**Table S11.** Appraisal of druggability.

**Additional file 2:**

STROBE-MR checklist
